# Sulfur Nanoparticle-Decorated Nickel Cobalt Sulfide Hetero-Nanostructures with Enhanced Energy Storage for High-Performance Supercapacitors

**DOI:** 10.3390/molecules27217458

**Published:** 2022-11-02

**Authors:** Yedluri Anil Kumar, Anuja A. Yadav, Bandar Ali Al-Asbahi, Seok-Won Kang, Md Moniruzzaman

**Affiliations:** 1 Department of Physics, United Arab Emirates University, Al Ain 15551, United Arab Emirates; 2 Department of Automotive Engineering, Yeungnam University, 280 Daehak-ro, Gyeongsan 38541, Gyeongbuk-do, Korea; 3 Department of Physics & Astronomy, College of Science, King Saud University, P.O. Box 2455, Riyadh 11451, Saudi Arabia; 4 Department of Chemical and Biological Engineering, Gachon University, 1342 Seongnam-daero, Seongnam-si 13120, Gyeonggi-do, Korea

**Keywords:** NiCo_2_S_4_, sulfur nanoparticle, electrode materials, supercapacitor, energy storage performance

## Abstract

Transition-metal sulfides exaggerate higher theoretical capacities and were considered a type of prospective nanomaterials for energy storage; their inherent weaker conductivities and lower electrochemical active sites limited the commercial applications of the electrodes. The sheet-like nickel cobalt sulfide nanoparticles with richer sulfur vacancies were fabricated by a two-step hydrothermal technique. The sheet-like nanoparticles self-combination by ultrathin nanoparticles brought active electrodes entirely contacted with the electrolytes, benefiting ion diffusion and charges/discharges. Nevertheless, defect engineers of sulfur vacancy at the atomic level raise the intrinsic conductivities and improve the active sites for energy storage functions. As a result, the gained sulfur-deficient NiCo_2_S_4_ nanosheets consist of good specific capacitances of 971 F g^−1^ at 2 A g^−1^ and an excellent cycle span, retaining 88.7% of the initial capacitance over 3500 cyclings. Moreover, the values of capacitance results exhibited that the fulfilling characteristic of the sample was a combination of the hydrothermal procedure and the surface capacitances behavior. This novel investigation proposes a new perspective to importantly improve the electrochemical performances of the electrode by the absolute engineering of defects and morphologies in the supercapacitor field.

## 1. Introduction

Worldwide, research is rising investigating safe, portable, renewable electrochemical electrical applications, and lightweight electrical gadgets, such as supercapacitors (SCs), which consider eco-friendly stabilities as well as higher energy/power densities [1,2,3]. In energy storage applications, supercapacitors (SCs) attained widespread consciousness on account of their extraordinary benefits in terms of rapid charging/discharging procedures, high reliability, excellent power densities, cheaper price, and environmental natures [4,5,6,7]. Meanwhile, the weaker energy densities of SCs have constricted them from laboratory scales to practical-span productivity [8,9,10]. Thus, higher performance sample-electrodes are worth finding for enhancing the energy densities of SCs.

Among them, transition-metal sulfides were advised as an essential source for the SCs application, owing to their higher capacity results, cheaper prices, innate abundances, and eco-friendliness [11]. Comparable to binary metal sulfides, bimetallic sulfide types (e.g., CuCo_2_S_4_ [12], NiCo_2_S_4_ [13], FeCo_2_S_4_ [14], MnCo_2_S_4_ [15]) normally manifest good added electrochemical behaviors for the given two causes: (i) the synergetic reaction of bimetallic sulfide types would consider a richer redox reaction; and (ii) bimetallic sulfide types normally exhibit higher conductivities [16,17,18]. Nevertheless, the insufficient energy storage active sites and weaker ion diffusion kinetics were reasoned by the limit of ion diffusion connections, which led to poorer rate capabilities and specific capacities [19]. Recent research has been maintained to enhance the electrochemical performances of the samples’ electrodes by morphology optimization and defect formations [20,21,22]. For instance, Wan et al. constructed NiCo_2_S_4_ porous nanotubes using sacrifices precursors to reason redox reactions at intrinsic activate sites, allow for faster ion transportations in the aqueous electrolyte, and represent a higher specific capacity of 933 F g^−1^ at 1 A g^−1^ [23]; Chen et al. fabricated caterpillar-type NiCoS crystals, exhibiting the higher specific capacitance of 1777 F g^−1^ at 1 A g^−1^ and retaining a 90.9% capacity of the starting cycles over 3000 cycles at 10 A g^−1^ [24]; Zhou et al. synthesized oxygen-deficient (V_o_-ZnO/CoO) nanowires [25], in which the oxygen defect improves the redox processes, allowing for faster electron transportation rates; and Huang et al. fabricated NiS@C with a sulfur vacancy (H-NiS_1−X_/C−50), where the sulfur vacancy formations provide lattice distortion reactions, exhibiting improved conductivities and good electrochemical activities (1728 F g^−1^, 1 A g^−1^) [26]. Evidentially, the combination of sulfur-deficient NiCo_2_S_4_ with a conductive matrix-type nickel foam can enhance the cycling performances and rate capabilities of materials. Meanwhile, it has been noted that bimetallic nickel cobalt sulfide consists of numerous redox sites and abundant valence states [27]; therefore, it might be a favoring electrode applicant for SCs, in which its less efficient ion/electron transportations hinder the electrochemical activity. To the best of our understanding, the performances of a sulfur-deficient NiCo_2_S_4_ were not adequate, and it was the main endeavor to further boost the electrochemical activities of SCs. Compared to the recently reported composited bimetallic sulfides, it could be anticipated that the sulfur-deficient NiCo_2_S_4_ composite might provide stronger cooperative effects and assist volume alterations, promising to enhance the electrochemical performances for SCs applications.

Based on the above findings, we integrate deficient engineering with rational nano-morphology construction to grow sheet-like nickel cobalt sulfide nanoparticles, self-assembling by ultrathin nanoparticles with richer sulfur vacancies through hydrothermal treatment. The fabricated electrode consists of higher conductivities and numerous abundant activation sites, providing good specific capacitances of 971 F g^−1^ at 2 A g^−1^ and the 88.7% retained rate of the prior capacitances over 3500 long cycles. Moreover, the sulfur-deficient NiCo_2_S_4_ nanosheets electrode exhibits lower resistances, revealing the extraordinary current conductivity of the sample material. In the investigation, the interconnected nanoparticle-type sulfur-deficient NiCo_2_S_4_ nanosheets composite was illustrated to be perfect for SCs applications.

## 2. Materials and Methods

### 2.1. Reagents and Chemicals

Nickel nitrate hexahydrate (Ni(NO_3_)_2_·6H_2_O, 99.0%), cobalt nitrate hexahydrate (Co(NO_3_)_2_·6H_2_O, 98.6%), and sodium sulfide nonahydrate (Na_2_S·9H_2_O, 98.0%] were purchased by Sigma-Aldrich. All the chemicals were analytically graded and used without any further procedure. The nickel foam was pre-treated before being utilized. 

### 2.2. Preparation of Sheet-like NiCo_2_S_4_ Nanoparticles with Richer Sulfur Vacancies on Ni Foam

In the preparation of sulfur-deficient sheet-like NiCo_2_S_4_ nanoparticles, 0.5 mmol Ni(NO_3_)_2_·6H_2_O, 0.9 mmol Co(NO_3_)_2_·6H_2_O, and 1.17 mmol Na_2_S·9H_2_O were dissolved in 40 mL of DI water under continuous stirring to gain a pink product. Next, the Ni foam with product precursors was kept in a Teflon-lined stainless autoclave and optimized at 110 °C for 13 h. After reaching room temperature, the samples were rinsed with DI water and subjected to the drying process to gain the needed products. After being dried, the black precipitate product was annealed at 400 °C for 1 h in a furnace in a continual Ar atmosphere. The representative mass load of the sulfur-deficient NiCo_2_S_4_ composite was ∼2.6 mg cm^−2^.

### 2.3. Characterizations and Instruments

The surface morphology and structures of the samples were observed using a scanning electron microscope (SEM, JSM-7800F equipping) and transmission electron microscopy (TEM, JEM-2100F, Busan, Korea). The crystal structures of the samples were determined using characteristic diffraction peaks measured by X-ray diffraction (XRD) analysis, which was performed using Bruker D8 Advance. X-ray photoelectron spectroscopy (ESCCALAB 250Xi, Busan, Korea) was organized to investigate the chemical bonding details of the electrodes.

### 2.4. Electrochemical Performance Test

The evaluation of all the electrochemical calculations for the electrodes was organized in 3: an electrode setup employing the current precursor products as the working electrodes, Platinum (Pt) sheet as the counter, and Ag/AgCl as the references electrode. Moreover, the current working electrode was assessed utilizing a 3-electrode cell with 2 M KOH electrolytes. Galvanostatic charge–discharge (GCD), cyclic voltammetry (CV), and electrochemical impedance spectroscopies (EIS) are evaluated by an electrochemical workstation. The specific capacitances (*C_sp_*, F g^−1^) of the samples were calculated by Equation (1) based on CD plots [28,29,30]:

*C*_s_ = (*I* × Δ*t*)/*m* × Δ*V* (1)

where Δ*t* (s), *I* (A), *m* (g), and Δ*V* (V) designate the discharge times, currents, the mass of the electrode, and the applied potential, respectively. 

## 3. Results and Discussion

### 3.1. Structure Characterization

**Figure 1:**
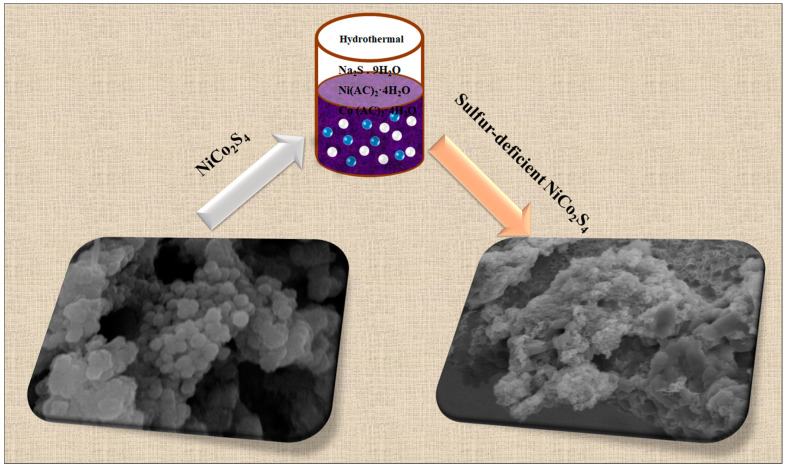
Schematic diagram illustrating the preparation of NiCo_2_S_4_ electrode and sulfur-deficient NiCo_2_S_4_ nanosheet composites on Ni foam substrate.

The fabrication of the sulfur-deficient sheet-like NiCo_2_S_4_ nanoparticles on nickel foam is reported in Figure 1. The Ni foam consists of a porous nature composed of interconnected nickel skeletons, which possess the benefits of excellent electrical conductivities and richer porosity, consequently supplying numerous active sites for the redox process. Thus, the growth of sheet-like NiCo_2_S_4_ particles on the nickel skeleton was a reasonable possibility. First, the NiCo_2_S_4_ particles were acquired by a hydrothermal process accompanied by annealing at 400 °C for 1 h in the Ar atmosphere. The gained thin nanosheets were collected from nanosheets with a porous nature.

**Figure 2:**
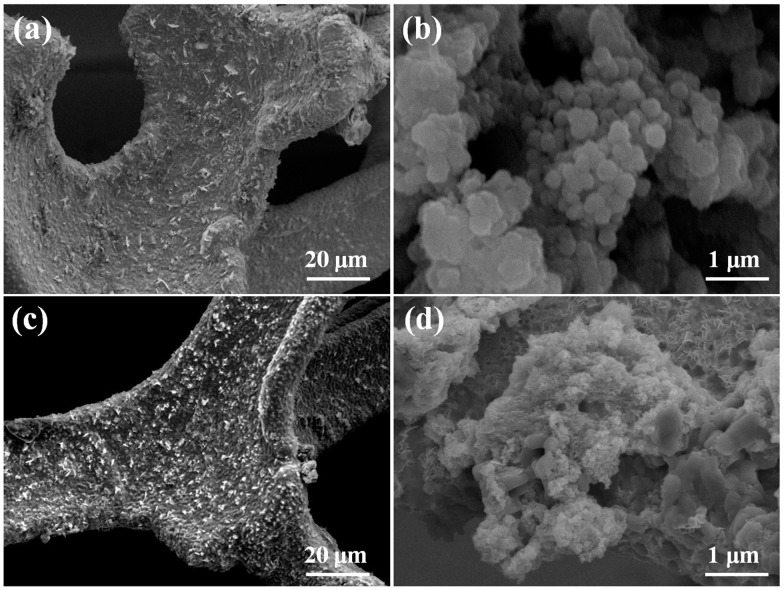
(**a,b**) SEM and FESEM images of pure NiCo_2_S_4_ nanoparticles electrode; and (**c,d**) SEM and FESEM images of the sulfur-deficient NiCo_2_S_4_ nanosheets composite.

Figure 2 displays the microscopic morphology of the as-synthesized pure NiCo_2_S_4_ nanoparticles electrode and sulfur-deficient NiCo_2_S_4_ nanosheet composite samples. As illustrated in Figure 2a,b, the pure NiCo_2_S_4_ nanoparticles sample holds slightly incomplete developed and shapeless particle-type morphologies. Dissimilar to the pure NiCo_2_S_4_ nanoparticles, the sulfur-deficient NiCo_2_S_4_ nanosheets composite was made up of ultrathin-type nanosheets, self-assembling sheet-like particles with a median diameter of around 7 μm (Figure 2c,d). This hierarchical nano-micro unique construction would supply abundant redox sites with higher active sites, condensing the diffusion networks of electrons/ions throughout the electrochemical reactions. In addition, the hydrothermal treatment affected the microstructure and crystallinity of the sulfur-deficient NiCo_2_S_4_ nanosheets composite, with the composite leading to the agglomeration of metallic nanoparticles and the electrochemical reactions of the electrode.

**Figure 3:**
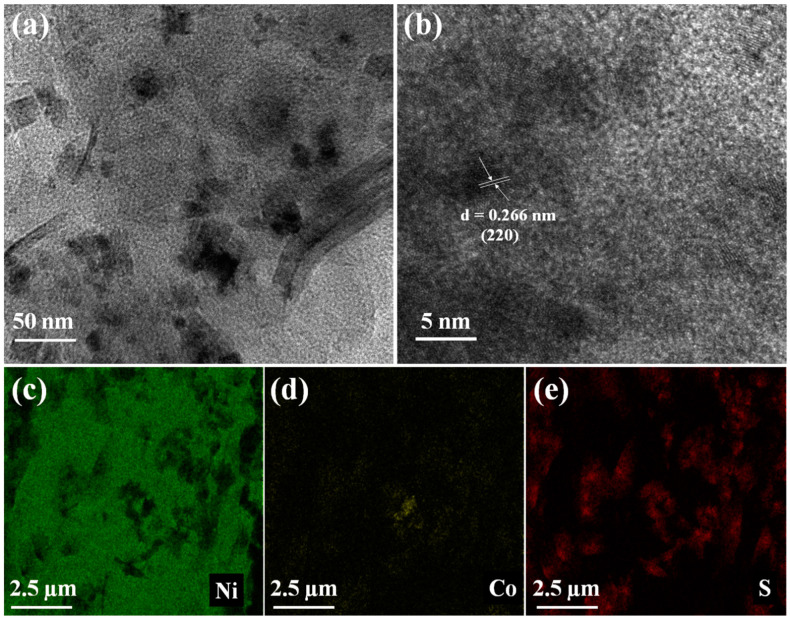
(**a,b**) TEM and HRTEM images, and (**c**–**e**) the elemental mapping images of sulfur-deficient NiCo_2_S_4_ nanosheets composite.

Figure 3 displayed the TEM and HRTEM images of sulfur-deficient NiCo_2_S_4_ nanosheet composites. TEM images in Figure 3a further clarified that the metallic particles were flat and immobilized on the nickel foam skeleton porous structure. Figure 3b shows that, in the high-resolution TEM images, sulfur-deficient NiCo_2_S_4_ nanosheet composites possess lattice fringes, such that spaces of 0.24 and 0.28 nm were acquired; which are in excellent accordance with the (400) and (511) planes of NiCo_2_S_4_, respectively [31,32]. On the other hand, the EDS elemental maps in Figure 3c–e depicted the uniform distributions of Ni, C, and S, revealing that the sulfur-deficient NiCo_2_S_4_ nanosheet composites are victoriously synthesized by a two-pot hydrothermal route.

**Figure 4:**
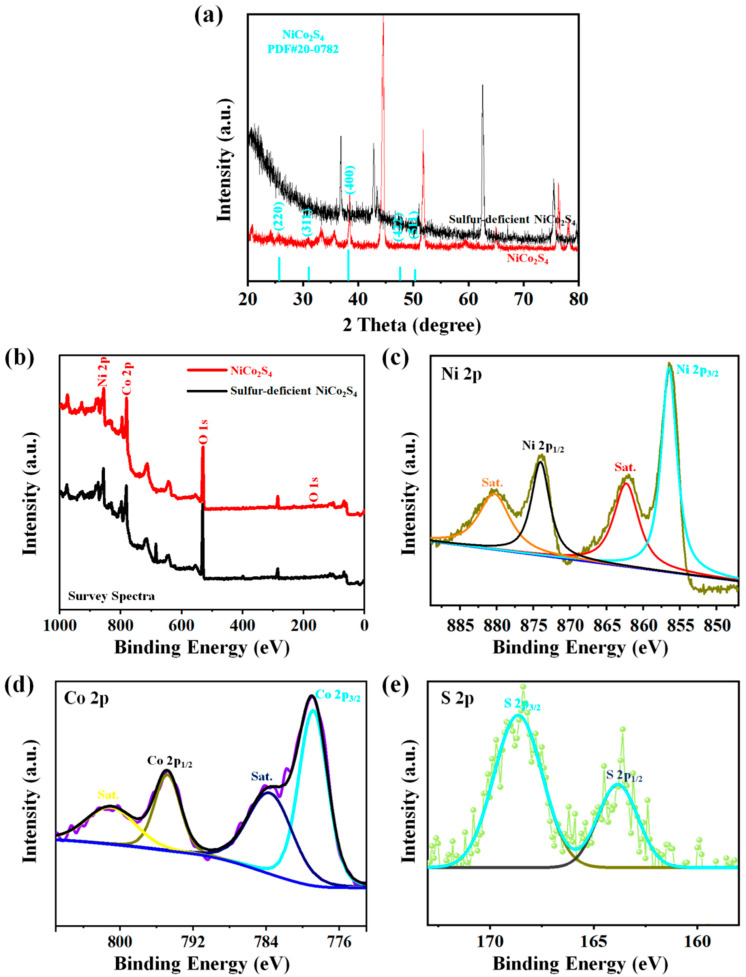
(**a**) XRD patterns of pure NiCo_2_S_4_ nanoparticles electrode and sulfur-deficient NiCo_2_S_4_ nanosheet composites. (**b**) XPS survey spectra, and (**c**–**e**) Ni 2p, Co 2p, and S 2p spectra for sulfur-deficient NiCo_2_S_4_ nanosheet composites.

The diffraction phase and purity of the as-reported pure NiCo_2_S_4_ nanoparticles electrode and sulfur-deficient NiCo_2_S_4_ nanosheet composites are investigated using X-ray diffraction analysis (Figure 4). The characteristic peaks at 26.7°, 31.5°, 38.4°, 47.5°, and 50.6° are attributed with the (220), (311), (400), (422), and (511) planes of the cubic NiCo_2_S_4_ phases, respectively (PDF#20-0782) [33,34]. It is obvious that all the good diffraction peaks of the NiCo_2_S_4_ would be ascribed to NiCo_2_S_4_ without clear diffractions correlated to the impurities, which evidences the successful preparation of sulfur-deficient NiCo_2_S_4_ nanosheet composites. Moreover, no additional peaks from the remaining crystallized planes could be gained from the electrode, noticing the excellent form of a pure NiCo_2_S_4_ sample.

**Figure 5:**
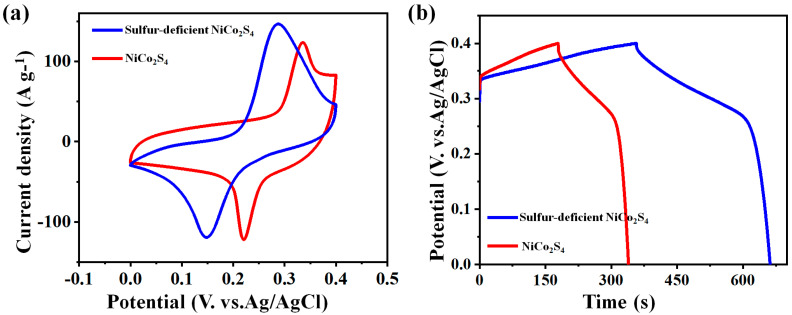
(**a**) CV curves and (**b**) GCD curves of pure NiCo_2_S_4_ nanoparticles electrode and sulfur-deficient NiCo_2_S_4_ nanosheet composites.

The chemical composition and elemental bonding states of the as-reported electrodes are fixed by using XPS spectra (Figure 4b–e). Figure 4b indicates a survey spectra of the sulfur-deficient NiCo_2_S_4_ nanosheets composites., which reports a dominance of Ni, C, and S [35]. The typical spectrum of Ni 2p (Figure 5b) and Co 2p (Figure 5c) corroborate the binary spin-orbits, respectively. In Figure 5b, in which there are the Ni 2p spectra, the 875.7 and 856.5 eV peaks are located from the Ni 2p_1/2_ and Ni 2p_3/2_, respectively [36,37]. Accordingly, the Co 2p spectra in Figure 5c illustrate that the 802.4 and 783.3 peaks are located therein and associated with Co 2p [38,39,40]. Figure 5d displays the XPS spectrum of S 2p, revealing the existence of sulfide ions in the binary Ni-Co-S. The foremost peaks of S 2p were allocated at 168.9 eV. These noticed data were related to the earlier reporting [41]. The XPS findings recognize that the atomic ratios of Ni, Co, and S elements in the NiCo_2_S_4_ nanosheets were 1:2.93:4.94, which correlates with the findings output of the NiCo_2_S_4_ samples. The XRD and XPS results were fitting, considering the chemical elemental states and the formation compositions of the pure NiCo_2_S_4_ phases. Furthermore, the XPS spectra displayed the mixed chemical elemental states necessity of Ni 2p/Ni 3p and Co 2p/Co 3p, which play an important role in developing the entire electrochemical activities of the NiCo_2_S_4_ sample.

### 3.2. Electrochemical Performance

The CV and GCD plots were first organized to find out the electrochemical activities of the pure NiCo_2_S_4_ nanoparticles electrode and the sulfur-deficient NiCo_2_S_4_ nanosheet composites in the three-electrode setup. As illustrated in Figure 5a, a symmetric Faraday redox pairs plot was noticed from the CV plot, which is because of their highly reversible redox process amid Ni^3+^/Ni^2+^ and Co^3+^/ Co^2+^ beneath alkaline solutions, resulting in the typical nature of PCs. The Faradaic possible reaction of the sulfur-deficient NiCo_2_S_4_ nanosheets composite was suggested as given [42,43]:

Ni_3_S_4_ + OH^−^ ↔ Ni_3_S_4_ (OH) + e^−^

Ni_3_S_4_ (OH) + OH^−^ ↔ Ni_3_S_4_O + H_2_O + e^−^

CoO_2_ + e^−^ + H_2_O ↔ Co^(3+)^ OOH + OH^−^

The CV plot region of the sulfur-deficient NiCo_2_S_4_ nanosheet composites was much larger than that of the pure NiCo_2_S_4_ nanoparticles electrode, revealing its high charging storing capability. Figure 5b illustrates the GCD plots of both electrodes, and clear charge/discharge platforms were denoted in all the particulars, further proposing its PCs properties. As expected, the sulfur-deficient NiCo_2_S_4_ nanosheet composites showed longer discharging times, which disclosed their higher capacitances.

**Figure 6:**
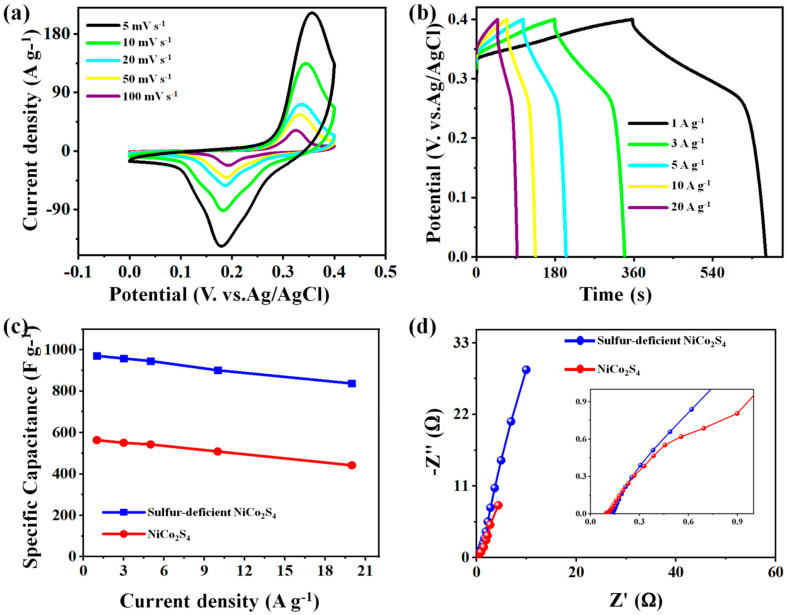
(**a**) CV curves and (**b**) GCD curves of the sulfur-deficient NiCo_2_S_4_ nanosheets composite electrode; (**c**) specific capacitances and (**d**) EIS curves of the pure NiCo_2_S_4_ nanoparticles electrode, and sulfur-deficient NiCo_2_S_4_ nanosheet composite electrodes.

Figure 6a discloses the CV plots of sulfur-deficient NiCo_2_S_4_ nanosheet composites at various sweep rates. Acquiescing with the polarizing effects of the sample electrodes, improving the sweep rates (5 to 100 mV s^−1^) led to the cathode’s peak shifting to fewer potentials, whereas the anode, peaking according to the reverse trends, moved to a higher potential. Figure 6b displays the GCD plot of the sulfur-deficient NiCo_2_S_4_ nanosheets composite electrode at different current values (2 to 20 A g^−1^). The plot shapes are nearly similar and symmetrical, indicating its good electrochemical reversibility. Figure S1 demonstrates the CV and GCD plots of the pure NiCo_2_S_4_ nanoparticles electrode at various scan rates (5 to 100 mV s^−1^) and current densities (2 to 20 A g^−1^) in the 2M KOH aqueous electrolyte; this further exhibited its superior rate capabilities. According to the GCD results, the measured specific capacitance of both electrodes was reported in Figure 6c. At 1 A/g, the specific capacitances of the pure NiCo_2_S_4_ nanoparticles electrode and sulfur-deficient NiCo_2_S_4_ nanosheet composites were 564 and 971 F g^−1^, respectively. These data values confirm that the hydrothermal route significantly overblows the specific capacitances of the composite. On the contrary, the higher sulfur-deficient NiCo_2_S_4_ nanosheets composite leads to the agglomeration of the metallic particle; thus, hindering the electrical migrations and holding back their electrochemical process. When the sulfur-deficient NiCo_2_S_4_ nanosheets composite is at 2 A g^−1^, a specific surface region is large enough to reveal the required electrochemical active speeds to enhance the networks of the electrode sample and electrolytes.

In Figure 6d, the pure NiCo_2_S_4_ nanoparticles electrode and sulfur-deficient NiCo_2_S_4_ nanosheet composites reported smaller semi-circled diameters in the higher-frequencies area, and have roughly vertical lines in the lower frequencies region; this hinted that the sulfur-deficient NiCo_2_S_4_ nanosheet composites have optimal conductivities as well as quick charging transportation capabilities. The EIS curve is composed of the equivalent series resistance (Rs), the charge transfer resistance (Rct), and Warburg diffusion resistance (R_w_), which is related to the resistance of ionic diffusion in the electrolyte. The fitting results show that the Rct values of the sulfur-deficient NiCo_2_S_4_ nanosheets composite and pure NiCo_2_S_4_ are 0.145 and 0.194 Ω, respectively. Therefore, it can be determined that the charge transfer resistance of the sulfur-deficient NiCo_2_S_4_ nanosheets composite is the smallest. Furthermore, the equivalent series resistance values of the sulfur-deficient NiCo_2_S_4_ nanosheets composite and pure NiCo_2_S_4_ are 1.732 and 0.831 Ω, respectively. The slope of the sulfur-deficient NiCo_2_S_4_ nanosheets composite is the largest, indicating that it has a high diffusivity of the electrolyte. In both cases, the Rs and Rct values of the sulfur-deficient NiCo_2_S_4_ nanosheets composite has a strong dynamic response and electrical conductivity.

**Figure 7:**
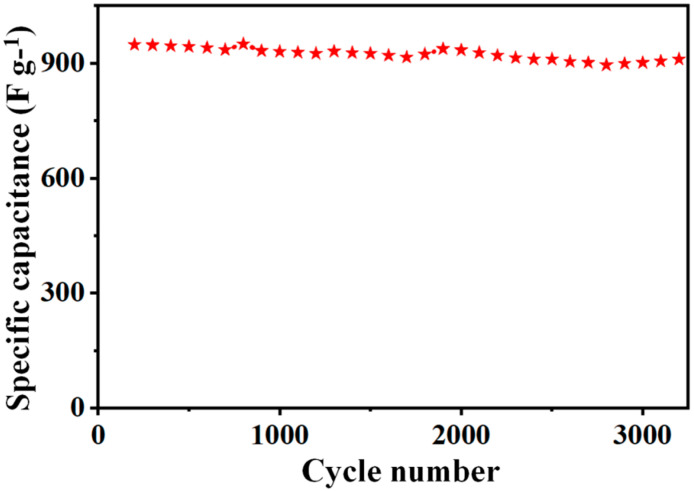
Cycling stability of the sulfur-deficient NiCo_2_S_4_ nanosheet composites over 3500 long cycles at a constant current density of 3 A g^−1^.

Figure 7 illustrates the long cycles of the sulfur-deficient NiCo_2_S_4_ nanosheet composites at 3 A g^−1^. After 3500 long cycles of charges/discharges, the capacity retention of the sulfur-deficient NiCo_2_S_4_ nanosheet composites is still retained at 88.7%, further illustrating the outstanding life span of cycling stability analysis. From Table 1, the specified capacitance results data of the electrodes are demonstrated for comparison. Figure S2 illustrates the EIS curves of the sulfur-deficient NiCo_2_S_4_ before and after 3500 cycles of the stability test at 3 Ag^−1^.

## 4. Conclusions

We demonstrated a higher-efficiency approach to enhance the higher-performance electrode materials for supercapacitors by constructing rational nanomorphologies and increasing sulfur vacancies in NiCo_2_S_4_. The physical morphology data results exhibited that the hydrothermal route corresponds to the composite material crystallinity. By the data values, the as-developed sulfur-deficient NiCo_2_S_4_ nanosheet composites display a superior specific capacitance of 971 F g^−1^ at 2 A g^−1^ and capacitance retentions of 88.7% at 3 A g^−1^ over 3500 long cycles. The excellent electrochemical capabilities would have corresponded to the involvement of sulfur vacancies and well-constructive superior specific area particle-like morphology that improves the electrode’s conductivities, enhances activated sites for the electrochemical reactions, and encourages the electrolyte penetrations into the sample material. This study would open unique and favoring paths for the succession of future generations of high-performance materials for SCs. The developed electrode exhibited attractive potential in SCs applications.

## Figures and Tables

**Table 1:** Comparison of the specific capacitance of the sulfur-deficient NiCo_2_S_4_ nanosheets composites electrode prepared in the present work and other reports in the literature.

Electrode	Preparation Method	Capacitance	Current Density	Ref.
NiCo_2_S_4_ hollow hexagonal nanoplates	Sacrificial template method	437 F g^−1^	1 A g^−1^	[44]
NiCo_2_S_4_ nanotube arrays	Hydrothermal method	738 F g^−1^	4 A g^−1^	[45]
NiCo_2_S_4_ nanoflakes	Ionic layer adsorption	1076 F g^−1^	1 A g^−1^	[46]
NiCo_2_S_4_ hollow nanoprisms	Sacrificial template method	895.2 F g^−1^	1 A g^−1^	[47]
NiCo_2_S_4_ nanotube arrays	Electrodeposition	2.86 F cm^−2^	4 mA cm^−2^	[48]
NiCo_2_S_4_ nanostructure	Precursor transformation method	1050 F g^−1^	2 A g^−1^	[49]
Sulfur-deficient NiCo_2_S_4_ nanosheets	Hydrothermal method	971 F g^−1^	2 A g^−1^	This Work

## Data Availability

No new data were created or analyzed in this study. Data sharing is not applicable to this article.

## Supplementary Materials

The following supporting information can be downloaded at: https://www.mdpi.com/article/10.3390/molecules27217458/s1, Figure S1: CV and GCD plots of pure NiCo_2_S_4_ nanoparticles electrode at various scan rates and current densities in aqueous electrolyte; Figure S2: EIS curves of the sulfur deficient NiCo_2_S_4_ before and after 3500 cycles of stability test at 3 Ag^−1^.
