# Supplementary material for: Sulfur Nanoparticle-Decorated Nickel Cobalt Sulfide Hetero-Nanostructures with Enhanced Energy Storage for High-Performance Supercapacitors
Source: Molecules. 2022 Nov 2;27(21):7458. doi: 10.3390/molecules27217458 (PMC9658846; doi:10.3390/molecules27217458)
Supplement: Supplementary file 1 [file molecules-27-07458-s001.zip › molecules-1957396-supplementary.pdf]

<u>**Supplementary Materials**</u>

Sulfur nanoparticle-decorated nickel cobalt sulfide hetero-nanostructures with enhanced energy storage for high-performance supercapacitors

Yedluri Anil Kumar[a,#], Anuja A. Yadav[b,#], Bandar Ali Al-Asbahi[c], Seok-Won Kang[b], Md Moniruzzaman[d,*]

[a] Department of Physics, United Arab Emirates University, Al Ain 15551, United Arab Emirates

[b] Department of Automotive Engineering, Yeungnam University, 280 Daehak-ro, Gyeongsan, Gyeongbuk 38541, Republic of Korea.

[c] Department of Physics & Astronomy, College of Science, King Saud University, P.O.Box 2455, Riyadh 11451, Saudi Arabia.

[d] Department of Chemical and Biological Engineering, Gachon University, 1342 Seongnam-daero, Seongnam-si, Gyeonggi-do 13120, Republic of Korea.

[#] **Equal author contribution**

***Corresponding Author**

E-mail: mani57chem@gachon.ac.kr

Tel: +82 10 3261 0692.

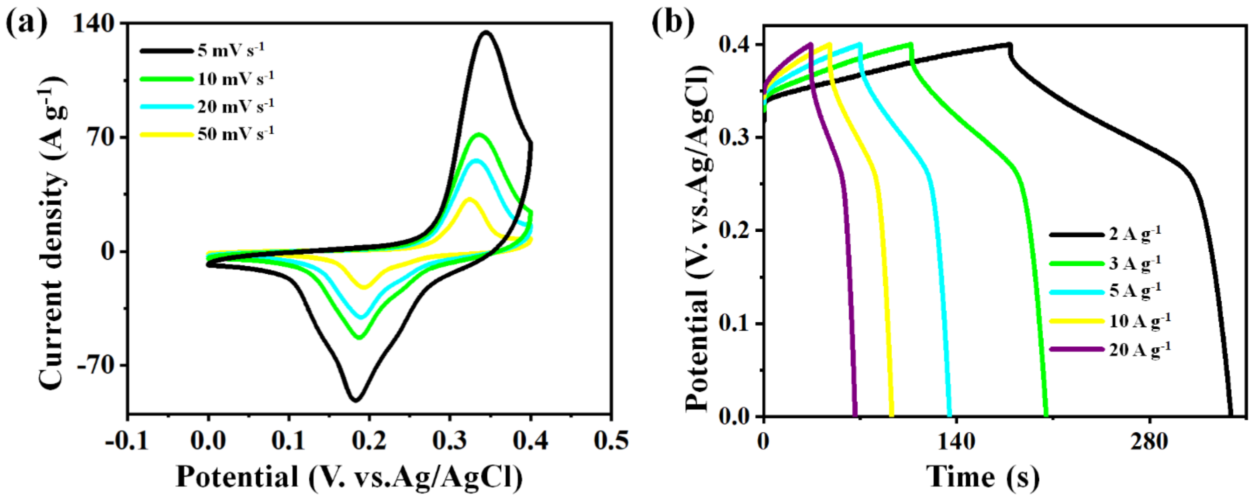

Figure S1. (a and b) CV and GCD plots of pure $NiCo_2S_4$ nanoparticles electrode at various scan rates and current densities in aqueous electrolyte.

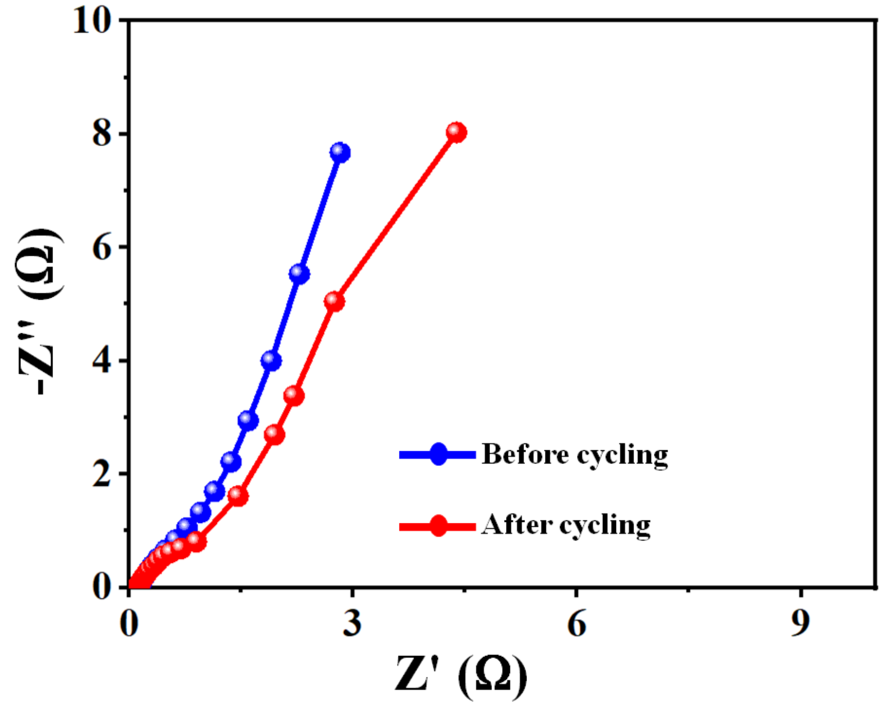

Figure S2. EIS curves of the sulfur deficient $NiCo_2S_4$ before and after 3500 cycles of stability test at 3 Ag^{-1}.
